# The clinical relevance of MOG antibody testing in cerebrospinal fluid

**DOI:** 10.1002/acn3.52163

**Published:** 2024-07-28

**Authors:** Molly Reynolds, Irene Tan, Kristy Nguyen, Vera Merheb, Fiona X. Z. Lee, Benjamin P Trewin, Magdalena Lerch, Snehal Shah, Nigel Wolfe, Katherine Buzzard, Jeannette Lechner‐Scott, Marzena Fabis‐Pedrini, Anthony Fok, Nevin John, Chris Kneebone, Con Yiannikas, David A. Brown, Allan G. Kermode, Stephen Reddel, Russell C. Dale, Fabienne Brilot, Sudarshini Ramanathan, the Australasian MOGAD Study Group, Robert Adam, Jane Andersen, Ian Andrews, Jayne Antony, Patrick Aouad, Monica Badve, Michael H. Barnett, Joshua Barton, Heidi Beadnall, Stefan Blum, Michael Boggild, Fabienne Brilot, Simon Broadley, David A. Brown, Jim Burrow, Helmut Butzkueven, Katherine Buzzard, Ann Bye, Anita Cairns, Sophie Calvert, Fiona Chan, Shabeed Chelakkadan, Melissa Chu, Damian R. Clark, Isabella Cotter, Russell C. Dale, Fionna Dela Cruz, Marzena J. Fabis‐Pedrini, Deborah Field, Anthony Fok, Clare L. Fraser, Victor S. C. Fung, Justin Garber, Serge Geara, Deepak Gill, Sachin Gupta, Todd A. Hardy, Simon Hawke, Andrew P. D. Henderson, Niroshan Jeyakumar, Nevin A. John, Dean L. Jones, Hannah F. Jones, Tomas Kalincik, Allan Kermode, Matthew Kiernan, Trevor Kilpatrick, Chris Kneebone, Andrew J. Kornberg, Mitchell Lawlor, Jeannette Lechner‐Scott, Fiona X. Z. Lee, Magdalena Lerch, Richard J. Leventer, Vivien Li, Simon Ling, Ganesha Liyanage, Joseph A. Lopez, Kit Kwan Margaret Ma, Stephen Malone, Mark P. Marriot, Pamela McCombe, Alan McDougall, Manoj P. Menezes, Vera Merheb, Christina Miteff, Mastura Monif, Gopinath Musuwadi Subramanian, Kristy Nguyen, Ai‐Lan Nguyen, Gina O'Grady, John O'Neill, Robert Ouvrier, Mark Paine, John Parratt, Sekhar Pillai, Jane Prosser, Jessica Qiu, Sudarshini Ramanathan, Stephen Reddel, Molly Reynolds, Sean DS Riminton, Izanne Roos, Jennifer Sandbach, Ingrid E. Scheffer, Snehal Shah, Ubaid Shah, Neil Shuey, Adriane Sinclair, Pakeeran Siriratnam, Mark Slee, Claire G. Spooner, Ian Sutton, Sanjay Swaminathan, Esther Tantsis, James Thomas, Terrence Thomas, Julia Thompson, Benjamin P. Trewin, Christopher Troedson, Anneke Van der Walt, Steve Vucic, Justine Wang, Tyson Ware, Richard Webster, Ming Wei Lin, Owen White, Nigel Wolfe, Wei Yeh, Con Yiannikas, Eppie M. Yiu, Michael Zhong

**Affiliations:** ^1^ Translational Neuroimmunology Group, Kids Neuroscience Centre, Faculty of Medicine and Health University of Sydney Sydney Australia; ^2^ Department of Neurology Concord Hospital Sydney Australia; ^3^ Department of Radiology Concord Hospital Sydney Australia; ^4^ Brain Autoimmunity Group Kids Neuroscience Centre, Children's Hospital at Westmead Sydney Australia; ^5^ Sydney Medical School and Brain and Mind Centre, Faculty of Medicine and Health University of Sydney Sydney Australia; ^6^ Department of Neurology, Perth Children's Hospital University of Western Australia Perth Australia; ^7^ Department of Neurology Blacktown Hospital Sydney Australia; ^8^ MS and Neuroimmunology Service, Royal Melbourne Hospital Melbourne Australia; ^9^ Hunter Medical Research Institute, Faculty of Medicine and Public Health University of Newcastle Newcastle Australia; ^10^ Department of Neurology John Hunter Hospital Newcastle Australia; ^11^ The Perron Institute for Neurological and Translational Science University of Western Australia Perth Australia; ^12^ The Centre for Molecular Medicine and Innovative Therapeutics Murdoch University Perth Australia; ^13^ Department of Neuroscience Monash Health Melbourne Australia; ^14^ Department of Medicine, School of Clinical Sciences Monash University Melbourne Australia; ^15^ Department of Neurology, Royal Adelaide Hospital Adelaide Australia; ^16^ Inner West Neurology Burwood Sydney Australia; ^17^ ICPMR and Department of Immunopathology, NSW Health Pathology Westmead Hospital Westmead Sydney Australia; ^18^ Clinical Immunology and Allergy Research Westmead Institute for Medical Research Westmead Sydney Australia; ^19^ Clinical Neuroimmunology Group Kids Neuroscience Centre, Children's Hospital at Westmead Sydney Australia; ^20^ TY Nelson Department of Neurology Children's Hospital at Westmead Sydney Australia; ^21^ School of Medical Science and Brain and Mind Centre, Faculty of Medicine and Health University of Sydney Sydney Australia

## Abstract

Myelin oligodendrocyte glycoprotein antibody‐associated disease (MOGAD) is diagnosed by serum MOG‐immunoglobulin G (MOG‐IgG) in association with typical demyelination. 111/1127 patients with paired CSF/serum samples were seropositive for MOG‐IgG. Only 7/1016 (0.7%) seronegative patients had CSF‐restricted MOG‐IgG. While 3/7 patients had longitudinally extensive transverse myelitis, four had a confirmed alternate diagnosis (three multiple sclerosis, one CNS vasculitis). In a national referral setting, CSF‐restricted MOG‐IgG had a low sensitivity (2.63%, 95%CI 0.55–7.50%) and low positive predictive value (1.97%, 95%CI 0.45–8.13%). We strongly recommend serum as the preferred diagnostic biospecimen, and urge caution in the interpretation of CSF‐restricted MOG‐IgG in patients without clinico‐radiological features consistent with MOGAD.

## Introduction

Myelin oligodendrocyte glycoprotein (MOG) antibody‐associated disease (MOGAD) is a demyelinating disorder that affects children and adults, and may be associated with recurrent or bilateral optic neuritis, acute disseminated encephalomyelitis, transverse myelitis, brainstem, cortical, or leptomeningeal involvement.^1^, ^2^, ^3^ Recently proposed diagnostic criteria highlight the importance of an appropriate clinical association, MOG antibody seropositivity by cell‐based assay, and exclusion of an alternate etiology.^3^ On occasion, testing reveals MOG‐immunoglobulin G (MOG‐IgG) in cerebrospinal fluid (CSF) without corresponding seropositivity (CSF‐restricted MOG‐IgG).^4^, ^5^, ^6^, ^7^ We report our experience in a national referral laboratory, and caution against the attribution of a diagnosis of MOGAD in unselected patients with a CSF‐restricted MOG‐IgG profile.

## Methods

We reviewed our national diagnostic database for unselected sequential samples of patients with CNS inflammation referred for MOG‐IgG testing by clinicians from the Australasian MOGAD Study Group and identified all patients with paired serum/CSF samples collected within 1 month of each other. Written informed consent and detailed clinical evaluation of patients with a CSF‐restricted MOG‐IgG profile was undertaken. MOG‐IgG was tested by a flow cytometry live cell‐based assay.^8^, ^9^ CSF was tested undiluted, serum at 1:50. Sensitivity, positive and negative predictive value (PPV, NPV), and number needed to predict (= 1/(PPV + NPV‐1)) were determined. Ethics approval was granted by the Sydney Children's Hospitals Network Human Ethics Committee (2019/ETH06041) and affiliated sites.

## Results

From 2018 to 2024, 1127 paired sera and CSF were tested. 111/1127 (9.85%) were seropositive for MOG‐IgG, with 59/111 (53%) additionally CSF MOG‐IgG positive. 1009/1016 (99.3%) seronegative patients were negative in paired CSF samples, while 7/1016 (0.7%) had a CSF‐restricted MOG‐IgG profile (Table 1). All seven patients were reproducibly MOG‐IgG seronegative, well below the threshold of detection, and not deemed to be “borderline”^9^ cases (Table 1). In all seven cases, sera tested was collected on the same day (4/7), 3 days prior (2/7) or 5 days prior (1/7) to CSF collection. Of these seven patients, four had an alternate diagnosis confirmed, including three with multiple sclerosis (MS) fulfilling 2017 Revised McDonald Criteria,^10^ and one with CNS vasculitis. We highlight two representative case reports below.

**Table 1:** Demographics, diagnoses, and cerebrospinal fluid (CSF) profile in CSF‐restricted MOG‐IgG patients.

Demographics			Clinical and/or radiological syndrome	Diagnosis	Cerebrospinal fluid analyses			
Case number	Age at onset (years)	Gender			RCC (×10^6^/L)	WCC (×10^6^/L)	Protein (g/L)	Oligoclonal bands
1	30	F	Unilateral optic neuritis with typical periventricular (Dawson's fingers) and juxtacortical white matter lesions	MS	48	0 PMNs, 4 monos	0.47	Intrathecally restricted
2	68	M	Multiterritory and multistaged ischemic infarctions and hemorrhage	CNS Vasculitis	450	90 PMNs, 56 monos	1.38	Not Detected
3	35	M	Typical periventricular and juxtacortical white matter lesions	MS	0	8 monos	0.51	Intrathecally restricted
4	33	M	Typical periventricular and juxtacortical white matter lesions	MS	3	0	NA	Intrathecally restricted
5	58	F	Longitudinally extensive transverse myelitis (monophasic)	Potential MOGAD phenotype	94	79 monos	0.63	Intrathecally restricted
6	52	F	Longitudinally extensive transverse myelitis; associated Sjogren's syndrome (monophasic)	Potential MOGAD Phenotype (DDx of Sjogren's TM)	17	4 monos	6.64	Not Detected
7	38	F	Longitudinally extensive transverse myelitis with follow‐up imaging revealing a section of cord necrosis; immunosuppressed prior to presentation for systemic lupus erythematosus (monophasic)	Potential phenotype for MOGAD (DDx of Parainfectious LETM)	10	1 PMN, 22 monos	0.63	Matched (present in serum and CSF)

CNS, central nervous system; CSF, cerebrospinal fluid; DDx, differential diagnosis; F, female; LETM, longitudinally extensive transverse myelitis; M, male; MOGAD, myelin oligodendrocyte glycoprotein antibody‐associated disease; monos, mononuclear cells; MS, multiple sclerosis; PMN, polymorphonuclear cell; RCC, red cell count; TM, transverse myelitis; WCC, white cell count.

### Case 1

**Figure 1:**
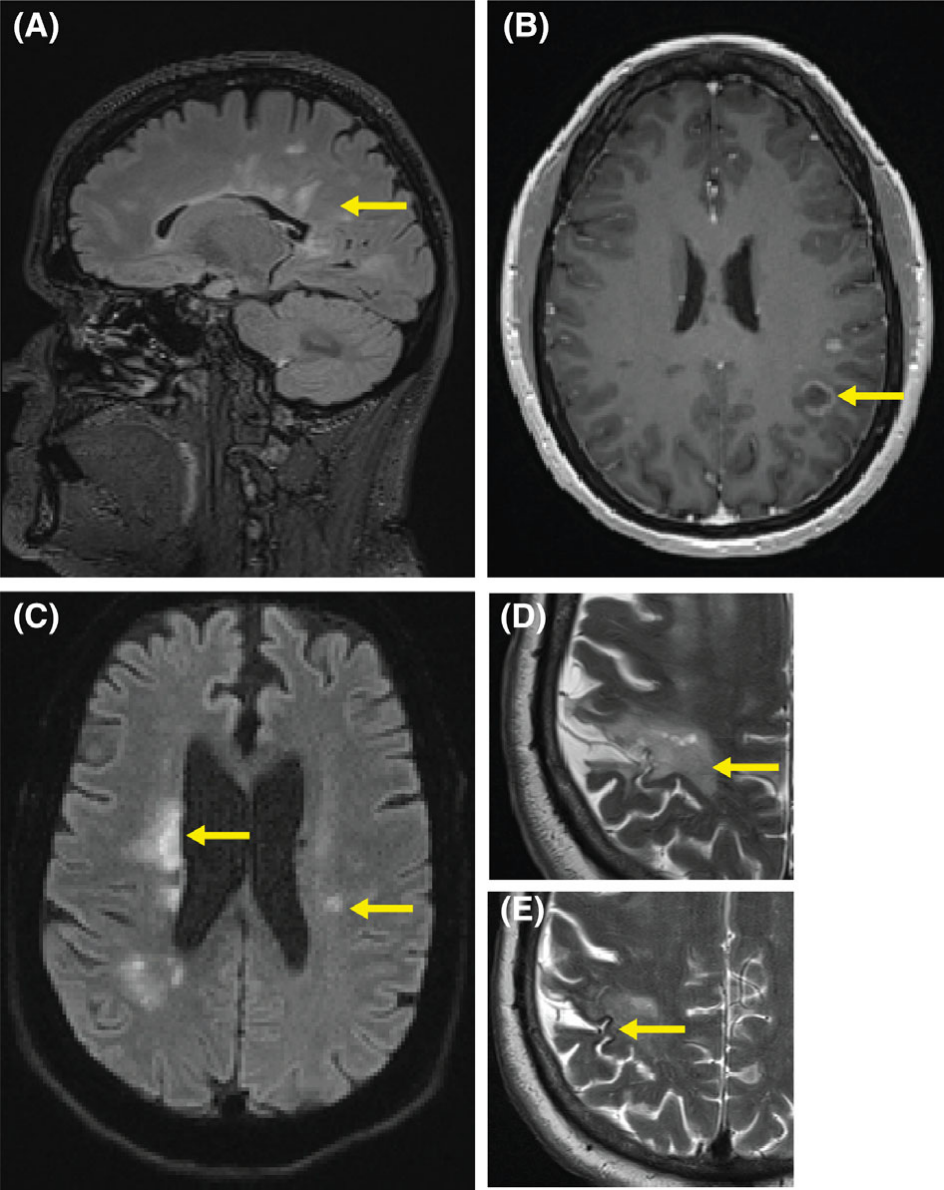
MRI features of non‐MOGAD diagnoses. (A) Perpendicular, periventricular T2 FLAIR hyperintensity (Dawson's Fingers, arrow) pathognomonic for multiple sclerosis. (B) T1 postgadolinium enhancement in an incomplete ring pattern (arrow) in the left parietal lobe consistent with demyelination in MS. (C) Bilateral periventricular and right cortical parietal lesions demonstrating diffusion restriction of varying intensity suggests recent established infarctions of varying acuity. (D and E) 3 months after (C) demonstrates right parietal lobe cortical and subcortical T2 hyperintensity with adjacent gyriform hemosiderin deposition consistent with interval established infarct and adjacent blood product deposition, in combination, suggestive of vasculitis.

A 30‐year‐old White female presented with bilateral upper and lower limb paresthesia, and historical symptoms of visual disturbance. Examination revealed right eye red desaturation and reduced visual acuity. MRI demonstrated radiological features typical for MS including perpendicular, periventricular lesions, and juxtacortical lesions with incomplete ring enhancement (Fig. 1A,B), and right optic nerve atrophy. Visual evoked potentials demonstrated prolonged latencies consistent with demyelination on the right. CSF analysis revealed elevated protein (0.47 g/L) with intrathecally restricted oligoclonal bands, 48 × 10^6^/L red cells, 4 × 10^6^/L mononuclear cells, and a CSF‐restricted MOG‐IgG profile. Her diagnosis was felt to be most consistent with MS, and she was commenced on natalizumab with prompt improvement in symptoms, before transitioning to ocrelizumab 5 months later due to positive John Cunningham virus serology. She remains clinically and radiologically quiescent at 4‐year follow‐up.

### Case 2

A 68‐year‐old Asian male presented with acute dysarthria. MRI demonstrated scattered multiterritory infarcts (Fig. 1C). He was commenced on maximal medical therapy. One month later, he suffered further multiterritory ischemic insults necessitating intensive care and intubation. MRI demonstrated multiple foci of ischemia in both left and right middle cerebral artery territories and the brainstem, with hemorrhage (Fig. 1D,E). CSF analyses demonstrated elevated protein (1.38 g/L), leukocytosis (90 × 10^6^/L polymorphonuclear cells, 56 × 106/L mononuclear cells), and red cells (450 × 10^6^/L). Intrathecally restricted oligoclonal bands were not detected. He had a CSF‐restricted MOG‐IgG profile. Cerebral angiography demonstrated multifocal vascular stenoses consistent with primary CNS vasculitis. Therapy was initiated with 5 days of intravenous methylprednisolone, followed by adalimumab, cyclophosphamide, and maintenance immunosuppression with mycophenolate. He was discharged following rehabilitation, with ongoing cognitive and functional limitations, but no further ischemic events in 12 months of follow‐up.

The remaining 3/7 patients (representing only 0.3% of seronegative patients tested) presented with monophasic longitudinally extensive transverse myelitis (LETM), classified as a potential phenotype for MOGAD. Of these three, one was felt to have a likely parainfectious myelitis as she was immunosuppressed for systemic lupus erythematosus, with no response to immunotherapy, and follow‐up imaging revealing a region of cord necrosis considered atypical for MOGAD. Another had transverse myelitis in the context of Sjogren's syndrome. Nevertheless in this study, to err on the side of caution, these three patients have been classified as “potential phenotypes for MOGAD,” as alternate diagnoses for their LETM were not definitively confirmed.

In the context of our national referral center, a CSF‐restricted MOG‐IgG profile had a low sensitivity and low positive predictive value (sensitivity: 2.63%, 95%CI 0.55–7.50%, PPV: 1.97%, 95%CI 0.45–8.13%) compared to serum testing. Consequently, 60 patients with CSF‐restricted MOG‐IgG would need to be examined, in order to correctly predict the diagnosis of one patient with a CSF‐restricted MOG‐IgG profile that is clinically relevant and consistent with a potential phenotype for MOGAD.

## Discussion

In most CNS autoimmune disorders with surface antigen‐directed autoantibodies, including MOGAD, serum autoantibody concentrations are consistently higher than CSF concentrations, reflecting their likely peripheral generation.^11^ Our study represents the largest unbiased assessment to date of sequential paired samples reflecting current referral practice, tested for MOG‐IgG in a national diagnostic center using a live cell‐based assay. This study highlights that a CSF‐restricted MOG‐IgG profile is rare, at a frequency of 0.7%. While some of these patients have a potential phenotype for MOGAD, our data show over half had an alternate diagnosis at follow‐up, reinforcing the need for caution in interpreting these results when tested in unselected patients.

An 11‐center Korean study evaluating paired serum and CSF of 474 patients with demyelination identified a CSF‐restricted MOG‐IgG profile in 4/31 (13%) patients with MS, and 9/217 (4%) patients with seronegative demyelination thought to be consistent with MOGAD.^5^ Among these patients with a potential phenotype for MOGAD, the CSF but not serum MOG‐IgG titer correlated with disability at attack.^5^ A CSF‐restricted MOG‐IgG profile was observed in 22/405 (5%) of patients with a potential phenotype for MOGAD in a Japanese cohort and 2/99 (2%) MS patients.^6^ The number needed to test for CSF‐restricted MOG‐IgG to diagnose one additional MOGAD case was 13.^6^ An international study evaluating paired serum/CSF in 255 selected patients with demyelination reported 31 (12%) patients had CSF‐restricted MOG‐IgG, with 27/31 thought to have a potential phenotype for MOGAD, associated with higher disability at follow‐up.^7^ 4/31 had alternate diagnoses of multiple sclerosis (*n* = 2), polyradiculoneuritis (*n* = 1), and Susac syndrome (*n* = 1). Analysis of children with MOG‐IgG reported 8% (9/109) had CSF‐restricted MOG‐IgG, associated with intrathecally restricted oligoclonal bands and MS.^12^ A recent study identified CSF MOG‐IgG positivity in 4/282 (1.4%) controls (MS, intracranial metastases, epilepsy, cord infarct), 66/74 (89%) seropositive MOGAD patients, and 9/73 (12%) seronegative patients with a potential MOGAD phenotype.^13^

Lower frequencies of CSF‐restricted MOG‐IgG have been reported in a national UK audit which identified that only 4/533 (0.8%) paired serum/CSF samples demonstrated CSF‐restricted MOG‐IgG^4^; with two presenting with LETM, a potential phenotype for MOGAD; and one with pneumococcal meningitis; and a number needed to test of 133 to identify one extra MOGAD patient.^4^ Our results similarly illustrate a low frequency of 0.7% of 1127 paired serum/CSF samples had this profile. Although all studies discussed above were performed utilizing a live cell‐based assay for MOG‐IgG detection, one possible reason for the reported discrepancy in frequencies may be due to differences in determining cutoffs for MOG‐IgG positivity between laboratories. Another contributor may be due to pre‐enrichment of patients from tertiary referral centers with potential phenotypes for MOGAD in some studies. In contrast, the UK study (0.8%)^4^ and our current results (0.7%) represent unbiased assessments of sequential samples tested in a national referral laboratory, which exemplifies the current referral practice of unselected testing for MOG‐IgG in paired samples from patients with any CNS inflammatory syndrome. The repercussions of unwarranted or inappropriately targeted immunosuppression and its associated adverse effects are notable.

Limitations of this study include a change in the positive threshold for serum and CSF MOG‐IgG detection from three to four standard deviations above the median fluorescence intensity of the control group.^9^ This represents a natural evolution in optimizing a diagnostic assay, and our center has validated highly specific and sensitive live cell‐based assays.^9^, ^14^ Importantly, all seven patients described in this case series maintained this profile even with optimized diagnostic cutoffs.

The pathogenic implications of intrathecally restricted MOG‐IgG are unclear. Patients with CNS inflammation may have intrathecal production of polyspecific antibodies against antigens such as MOG which are more accessible during active inflammation, as seen in MS.^15^, ^16^ Indeed, all studies to date have identified CSF‐restricted MOG‐IgG in some patients with MS. A recent study has shown that some CSF‐restricted MOG‐IgG cases have intrathecal synthesis of MOG‐IgG, rather than passive diffusion from the blood to intrathecal space, and that this might associate with disease severity.^17^ Additionally, the role of destructive inflammation of CNS tissue from any etiology and the relevance of nonpathogenic autoantibodies to exposed antigens should be considered.

Based on our results from the largest evaluation of paired serum/CSF samples to date, we recommend serum as the preferred biospecimen for testing MOG‐IgG. We do not recommend routine testing of CSF MOG‐IgG in unselected patients with diverse CNS inflammatory syndromes. If CSF is to be tested for MOG‐IgG, we recommend this be performed in seronegative patients with a phenotype suggestive for MOGAD. We advise caution in the interpretation of CSF‐restricted MOG‐IgG, as our data highlight that an alternate diagnosis was identified in over half the cases. This study supports the recent diagnostic criteria, which recommends additional clinico‐radiological features consistent with MOGAD and the careful exclusion of alternate etiologies are required to make a diagnosis of MOGAD in patients with a CSF‐restricted MOG‐IgG profile.^3^

## Conflict of Interest

MR has received travel support from Alexion and Roche and honoraria for an invited educational session from Novartis. BT receives a University of Sydney postgraduate scholarship and a stipend from CIA Ramanathan's Royal Australasian College of Physicians Research Establishment Fellowship. JL‐S received travel compensation from Biogen, Merck, and Novartis; has been involved in clinical trials with Biogen, Novartis, and Roche; her institution has received honoraria for talks and advisory board service from Biogen, Merck, Novartis, and Roche. She is on the board of directors for MSPlus. MF‐P has received a research grant from MS Australia and travel compensation from Merck. AGK has in recent times received speaker honoraria and Scientific Advisory Board fees from Bayer, BioCSL, Biogen‐Idec, Lgpharma, Merck, Novartis, Roche, Sanofi‐Aventis, Sanofi‐Genzyme, Teva, NeuroScientific Biopharmaceuticals, Innate Immunotherapeutics, and Mitsubishi Tanabe Pharma. His work has received grant funding from the Eyewall Foundation, Trish MS Foundation, MS Australia, MS Western Australia, the MS Base Foundation, the National Health and Medical Research Council of Australia, and the National Multiple Sclerosis Society, USA. He is an investigator in clinical trials sponsored by Biogen Idec and Novartis. NAJ is a principal investigator on commercial MS trials sponsored by Roche, Novartis, and Biogen. He has received speakers honoraria from Merck and travel congress sponsorship from Novartis. SWR has received travel support, honoraria, trial payments, research and clinical support to the neurology department or academic projects from NHMRC, MRFF, NBA, Myasthenia Alliance Australia, Lambert Initiative, Beeren foundation, anonymous donors; and from pharmaceutical/biological companies: Alexion, Biogen, CSL, Genzyme, Grifols, Merck, Novartis, Roche, Sandoz, Sanofi, and UCB. He is Co‐founder/shareholder of RxPx health, National IVIG Governance Advisory Council & Specialist Working Group Australia (Neurology) (paid), Australian Medical Services Advisory Committee ad hoc sub‐committee on IVIG (paid), Australian Technical Advisory Group on Immunisation Varicella Zoster working party (unpaid), Medical advisor (unpaid) to various patient and advocacy groups. Funds over the last 5 years including but not limited to travel support, honoraria, trial payments, research and clinical support to the neurology department or academic projects from: NHMRC, MRFF, NBA, Myasthenia Alliance Australia, Lambert Initiative, Beeren foundation, and anonymous donors; and from pharmaceutical/biological companies: Alexion, Biogen, CSL, Genzyme, Grifols, Merck, Novartis, Roche, Sandoz, Sanofi, and UCB. RCD has received research funding from the Star Scientific Foundation, The Trish Multiple Sclerosis Research Foundation, Multiple Sclerosis Research Australia, the Petre Foundation, and the NHMRC (Australia; Investigator Grant). He has also received honoraria from Biogen Idec as an invited speaker, and is on the IDMC for a Roche RCT in pediatric MS. He is on the medical advisory board (nonremunerated position). FB has received research funding from NSW Health, MS Australia, the NHMRC (Australia), the Medical Research Future Fund (Australia), The MOG Project (Apollo Grant) and Novartis. She was on an advisory board for Novartis and Merck, and has been an invited speaker for Biogen, Novartis, and Limbic Neurology. She is on the medical advisory board (nonremunerated positions) of The MOG Project and the Sumaira Foundation. SR has received research funding from the National Health and Medical Research Council (NHMRC, Australia), the Petre Foundation, the Brain Foundation, the Royal Australasian College of Physicians, and the University of Sydney. She is supported by an NHMRC Investigator Grant (GNT2008339). She serves as a consultant on an advisory board for UCB and Limbic Neurology, and has been an invited speaker for educational/research sessions coordinated by Biogen, Alexion, Novartis, Excemed, and Limbic Neurology. She is on the medical advisory board (nonremunerated positions) of The MOG Project and the Sumaira Foundation. All other authors have no relevant disclosures to report.

## Author Contributions

MR, FB, and SR had full access to all of the data in the study, and FB and SR are responsible for the overall content as guarantors. MR, FB, and SR contributed to the conception and design of the study, drafting a significant portion of the manuscript or figures, and statistical analyses. All authors contributed to acquisition and analysis of data, and interpretation of data; and critical review of the manuscript for important intellectual content. SR obtained funding and supervised MR. Authors from the Australasian MOGAD Study Group have contributed to data acquisition and analysis, and interpretation of data, and a full list of Study Group members are provided in the supplementary file.

## Funding Information

Study funding was provided by A/Professor Ramanathan's research grants including an NHMRC Investigator Grant (GNT2008339), Royal Australasian College of Physicians Research Establishment Fellowship, and University of Sydney grants.

## Supporting information


Table S1.

## Data Availability

Deidentified clinical data and samples can be provided upon reasonable request.
