# Supplementary material for: The clinical relevance of MOG antibody testing in cerebrospinal fluid
Source: Ann Clin Transl Neurol. 2024 Jul 28;11(9):2514–9. doi: 10.1002/acn3.52163 (PMC11537122; doi:10.1002/acn3.52163)
Supplement: Supplementary file 1 — Table S1. [file ACN3-11-2514-s001.docx]

**Australasian MOGAD Study Group Members**

| **Surname** | **First Name and Middle Initial(s)** | **Email address** | **Institution** | **Location (city, country)** |
| --- | --- | --- | --- | --- |
| Adam | Robert | robert.adam@health.qld.gov.au | Centre for Clinical Research - University of Queensland | Brisbane, Australia |
| Andersen | Jane | jane.andersen@sydney.edu.au | Translational Neuroimmunology Group, Kids Neuroscience Centre, Children's Hospital at Westmead, Sydney, New South Wales, Australia. | Sydney, Australia |
| Andrews | Ian | ian.andrews@health.nsw.gov.au | Department of Neurology, Sydney Children's Hospital | Sydney, Australia |
| Antony | Jayne | jayne.antony@health.nsw.gov.au | Department of Neurology, The Children's Hospital at Westmead | Sydney, Australia |
| Aouad | Patrick | patrick.aouad@sydney.edu.au | Sydney Medical School, Faculty of Medicine and Health, University of Sydney | Sydney, Australia |
| Badve | Monica | monica.badve@health.nsw.gov.au | Department of Neurology, St George Hospital | Sydney, Australia |
| Barnett | Michael H | michael@sydneyneurology.com.au | Brain and Mind Centre, Faculty of Medicine and Health and Brain and Mind Centre, University of Sydney | Sydney, Australia |
| Barton | Joshua | joshua@sydneyneurology.com.au | Department of Neurology, Sunshine Coast University Hospital | Birtinya, Australia |
| Beadnall | Heidi | heidi@sydneyneurology.com.au | Brain and Mind Centre, Faculty of Medicine and Health and Brain and Mind Centre, University of Sydney | Sydney, Australia |
| Blum | Stefan | stefan.blum@brisbaneneurology.com.au | Department of Neurology, Princess Alexandra Hospital | Brisbane, Australia |
| Boggild | Michael | mike.boggild@health.qld.gov.au | Townsville Neurosciences | Townsville, Australia |
| Brilot | Fabienne | fabienne.brilot@sydney.edu.au | Brain Autoimmunity Group, Kids Neuroscience Centre, Children's Hospital at Westmead | Sydney, Australia |
| Broadley | Simon | simon.broadley@griffith.edu.au | School of Medicine, Griffith University, Gold Coast, Queensland, Australia | Gold Coast, Australia |
| Brown | David A | david.brown1@sydney.edu.au | Westmead Institute for Medical Research & NSW Health Pathology-Westmead | Sydney, Australia |
| Burrow | Jim | jim.burrow@nt.gov.au | Department of Neurology, Royal Darwin Hospital | Darwin, Australia |
| Butzkueven | Helmut | helmut.butzkueven@monash.edu | Multiple Sclerosis and Neuroimmunology Research Groups, Department of Neuroscience, Monash University | Melbourne, Australia |
| Buzzard | Katherine | kabuzzard@gmail.com | Department of Neurology, Eastern Health | Melbourne, Australia |
| Bye | Ann | annie.bye@health.nsw.gov.au | Department of Neurology, Sydney Children's Hospital | Sydney, Australia |
| Cairns | Anita | anita.cairns@health.qld.gov.au | Department of Neurosciences, Queensland Children's Hospital | Brisbane, Australia |
| Calvert | Sophie | sophie.calvert@health.qld.gov.au | Department of Neuroscience, Queensland Children's Hospital | Brisbane, Australia |
| Chan | Fiona | [Fiona.chan12@outlook.com](mailto:Fiona.chan12@outlook.com) | Translational Neuroimmunology Group, Faculty of Medicine and Health, University of Sydney | Sydney, Australia |
| Chelakkadan | Shabeed | shabeed.chelakkadan@monashhealth.org | Department of Neurology, Monash Health | Melbourne, Australia |
| Chu | Melissa | melissa.chu@mh.org.au | CORe, Department of Medicine, MS Centre, The University of Melbourne | Melbourne, Australia |
| Clark | Damian R | damianr.clark@sa.gov.au | Department of Neurology, Women's and Children's Hospital | Adelaide, Australia |
| Cotter | Isabella | isabella.cotter@sydney.edu.au | Translational Neuroimmunology Group, Faculty of Medicine and Health, University of Sydney | Sydney, Australia |
| Dale | Russell C | russell.dale@health.nsw.gov.au | Clinical Neuroimmunology Group, Kids Neuroscience Centre, Children's Hospital at Westmead | Sydney, Australia |
| Dela Cruz | Fionna | fionna.delacruz@mh.org.au | CORe, Department of Medicine, MS Centre, The University of Melbourne | Melbourne, Australia |
| Fabis-Pedrini | Marzena J | marzena.pedrini@perron.uwa.edu.au | Perron Institute for Neurological and Translational Science, UWA Medical School, University of Western Australia | Perth, Australia |
| Field | Deborah | deborah.field@sa.gov.au | Department of Neurology, Royal Adelaide Hospital | Adelaide, Australia |
| Fok | Anthony | anthony.fok@monashhealth.org | Department of Neurology, Monash Health | Melbourne, Australia |
| Fraser | Clare L | clare.fraser@sydney.edu.au | Save Sight Institute, University of Sydney | Sydney, Australia |
| Fung | Victor SC | victor.fung@sydney.edu.au | Department of Neurology, Westmead Hospital | Sydney, Australia |
| Garber | Justin | justin.garber@health.nsw.gov.au | Department of Neurology, Westmead Hospital | Sydney, Australia |
| Geara | Serge | sgea3455@uni.sydney.edu.au | Translational Neuroimmunology Group, Kids Neuroscience Centre, Children's Hospital at Westmead, Sydney, New South Wales, Australia. | Sydney, Australia |
| Gill | Deepak | deepak.gill@health.nsw.gov.au | Children's Hospital at Westmead Clinical School, Faculty of Medicine and Health, University of Sydney | Sydney, Australia |
| Gupta | Sachin | sachin.gupta@sydney.edu.au | Department of Neurology, The Children's Hospital at Westmead, The University of Sydney | Sydney, Australia |
| Hardy | Todd A | thar6109@sydney.edu.au | Department of Neurology, Concord Repatriation General Hospital | Sydney, Australia |
| Hawke | Simon | simon.hawke@sydney.edu.au | Sydney Medical School, Faculty of Medicine and Health, University of Sydney | Orange, Australia |
| Henderson | Andrew PD | andrew.henderson@health.nsw.gov.au | Department of Neurology, Westmead Hospital | Sydney, Australia |
| Jeyakumar | Niroshan | niroshan.jeyakumar@health.nsw.gov.au | Translational Neuroimmunology Group, Kids Neuroscience Centre, Children's Hospital at Westmead, Sydney, New South Wales, Australia. | Sydney, Australia |
| John | Nevin A | [nevin.john@monashhealth.org](mailto:nevin.john@monashhealth.org) | Department of Medicine, School of Clinical Sciences, Monash University | Melbourne, Australia |
| Jones | Dean L | dean.jones@utas.edu.au | School of Medicine, University of Tasmania | Hobart, Australia |
| Jones | Hannah F | hannahj@adhb.govt.nz | Paediatric Neuroservices, Starship Hospital, Department of Paediatrics, University of Auckland | Auckland, New Zealand |
| Kalincik | Tomas | tomas.kalincik@unimelb.edu.au | CORe, Department of Medicine, MS Centre, The University of Melbourne | Melbourne, Australia |
| Kermode | Allan | kermode@me.com | Centre for Neuromuscular and Neurological Disorders, Western Australian Neuroscience Research Institute, University of Western Australia | Perth, Australia |
| Kiernan | Matthew | matthew.kiernan@sydney.edu.au | Department of Neurology, Royal Prince Alfred Hospital | Sydney, Australia |
| Kilpatrick | Trevor | tkilpat@unimelb.edu.au | Department of Medicine, MS Centre, The University of Melbourne, | Melbourne, Australia |
| Kneebone | Chris | ckneebon@bigpond.net.au | Department of Neurology, Royal Adelaide Hospital | Adelaide, Australia |
| Kornberg | Andrew J | andrew.kornberg@rch.org.au | Department of Neurology, The Royal Children's Hospital Melbourne | Melbourne, Australia |
| Lawlor | Mitchell | mitchell.lawlor@health.nsw.gov.au | Department of Neuro-ophthalmology, Sydney Eye Hospital | Sydney, Australia |
| Lechner-Scott | Jeannette | jeannette.lechnerscott@health.nsw.gov.au | Hunter Medical Research Institute, Faculty of Medicine and Public Health, University of Newcastle, Department of Neurology, John Hunter Hospital | Newcastle, Australia |
| Lee | Fiona XZ | fiona.zheng@health.nsw.gov.au | Brain Autoimmunity Group, Kids Neuroscience Centre, Children's Hospital at Westmead | Sydney, Australia |
| Lerch | Magdalena | Magdalena.Lerch@health.nsw.gov.au | Translational Neuroimmunology Group, University of Sydney | Sydney, Australia |
| Leventer | Richard J | richard.leventer@rch.org.au | Royal Children’s Hospital Department of Neurology, Murdoch Children’s Research Institute and University of Melbourne Department of Paediatrics | Melbourne, Australia |
| Li | Vivien | vivien.li@unimelb.edu.au | The Florey Institute of Neuroscience and Mental Health, University of Melbourne | Melbourne, Australia |
| Ling | Simon | simon.ling@singhealth.com.sg | Neurology Service Department of Paediatrics KK Women’s & Children’s Hospital Singapore | Singapore, Singapore |
| Liyanage | Ganesha | ganesha.liyanage@sydney.edu.au | Brain Autoimmunity Group, Kids Neuroscience Centre, Children's Hospital at Westmead | Sydney, Australia |
| Lopez | Joseph A | joseph.lopez@sydney.edu.au | Brain Autoimmunity Group, Kids Neuroscience Centre, Children's Hospital at Westmead | Sydney, Australia |
| Ma | Kit Kwan Margaret | kitkwanmargaret.ma@mh.org.au | CORe, Department of Medicine, MS Centre, The University of Melbourne | Melbourne, Australia |
| Malone | Stephen | stephen.malone@health.qld.gov.au | Department of Neuroscience, Queensland Children's Hospital | Brisbane, Australia |
| Marriot | Mark P | mark.marriott@mh.org.au | Department of Neurology, Royal Melbourne Hospital | Melbourne, Australia |
| McCombe | Pamela | pamela.mccombe@uq.edu.au | Department of Neurology, Royal Brisbane Hospital | Brisbane, Australia |
| McDougall | Alan | alan.mcdougall@health.nsw.gov.au | Department of Neurology, Liverpool Hospital | Sydney, Australia |
| Menezes | Manoj P | manoj.menezes@health.nsw.gov.au | Children's Hospital at Westmead Clinical School, Faculty of Medicine and Health, University of Sydney | Sydney, Australia |
| Merheb | Vera | vera.merheb@health.nsw.gov.au | Brain Autoimmunity Group, Kids Neuroscience Centre, Children's Hospital at Westmead | Sydney, Australia |
| Miteff | Christina | christina.miteff@health.nsw.gov.au | Department of Neurology, John Hunter Children's Hospital | Newcastle, Australia |
| Monif | Mastura | mastura.monif@monash.edu | Department of Neurology, Monash Health | Melbourne, Australia |
| Musuwadi Subramanian | Gopinath | gopinath.subramanian@health.nsw.gov.au | Department of Neurology, John Hunter Children's Hospital | Newcastle, Australia |
| Nguyen | Kristy | Kristy.Nguyen1@health.nsw.gov.au | Brain Autoimmunity Group, University of Sydney | Sydney, Australia |
| Nguyen | Ai-Lan | ai-lan.nguyen@unimelb.edu.au | CORe, Department of Medicine, MS Centre, The University of Melbourne | Melbourne, Australia |
| O’Grady | Gina | ginao@adhb.govt.nz | Paediatric Neuroservices, Starship Children's Health, Auckland District Health Board | Auckland, New Zealand |
| O'Neill | John | jhoneill@stvincents.com.au | Department of Neurology, St Vincent’s Hospital | Sydney, Australia |
| Ouvrier | Robert | robert.ouvrier@sydney.edu.au | Department of Neurology, The Children's Hospital at Westmead, The University of Sydney | Sydney, Australia |
| Paine | Mark | markpaine@me.com | Dept Neurology, Royal Brisbane & Womens Hospital | Brisbane, Australia |
| Parratt | John | john.parratt@sydney.edu.au | Department of Neurology, Royal North Shore Hospital | Sydney, Australia |
| Pillai | Sekhar | sekhar.pillai@health.nsw.gov.au | Department of Neurology, Sydney Children's Hospital | Sydney, Australia |
| Prosser | Jane | jane.prosser@health.nsw.gov.au | Department of Neurology, St George Hospital | Sydney, Australia |
| Qiu | Jessica | jessica.qiu@health.nsw.gov.au | Translational Neuroimmunology Group, Kids Neuroscience Centre, Children's Hospital at Westmead, Sydney, New South Wales, Australia. | Sydney, Australia |
| Ramanathan | Sudarshini | sudarshini.ramanathan@sydney.edu.au | Translational Neuroimmunology Group, Kids Neuroscience Centre, Children's Hospital at Westmead, Sydney, New South Wales, Australia. | Sydney, Australia |
| Reddel | Stephen | swreddel@sydneyneurology.com.au | Department of Neurology, Concord Repatriation General Hospital | Sydney, Australia |
| Reynolds | Molly | Molly.Reynolds@health.nsw.gov.au | Department of Neurology, Concord Repatriation General Hospital | Sydney, Australia |
| Riminton | Sean DS | sean.riminton@sydney.edu.au | Department of Immunology, Concord Repatriation General Hospital | Sydney, Australia |
| Roos | Izanne | izanne.roos@mh.org.au | CORe, Department of Medicine, MS Centre, The University of Melbourne | Melbourne, Australia |
| Sandbach | Jennifer | jennifer.sandbach@health.nsw.gov.au | Department of Ophthalmology, Prince of Wales Hospital, University of New South Wales | Sydney, Australia |
| Scheffer | Ingrid E | i.scheffer@unimelb.edu.au | Department of Medicine and Paediatrics, The University of Melbourne, Austin and Royal Children’s Hospitals | Melbourne, Australia |
| Shah | Snehal | Snehal.Shah@health.wa.gov.au | Perth Paediatrics | Perth, Australia |
| Shah | Ubaid | ubaid.shah@health.qld.gov.au | Neurosciences Unit, Queensland Children's Hospital | Brisbane, Australia |
| Shuey | Neil | neil.shuey@eyeandear.org.au | Department of Neurology, St Vincent’s Hospital Melbourne, Royal Victorian Eye & Ear Hospital | Melbourne, Australia |
| Sinclair | Adriane | adriane.sinclair@health.qld.gov.au | Department of Neurosciences, Queensland Children's Hospital | Brisbane, Australia |
| Siriratnam | Pakeeran | p.siriratnam@alfred.org.au | Multiple Sclerosis and Neuroimmunology Research Groups, Department of Neuroscience, Monash University | Melbourne, Australia |
| Slee | Mark | mark.slee@flinders.edu.au | Flinders University | Adelaide, Australia |
| Spooner | Claire G | cspooner@adhb.govt.nz | Paediatric Neuroservices, Starship Children's Health, Auckland District Health Board | Auckland, New Zealand |
| Sutton | Ian | iansutton@hotmail.com | Department of Neurology, St Vincent’s Hospital | Sydney, Australia |
| Swaminathan | Sanjay | sanjay.swaminathan@sydney.edu.au | Department of Medicine, Western Sydney University and University of Sydney, Dept of Immunology, Western Sydney Local Health District | Sydney, Australia |
| Tantsis | Esther | esther.tantsis@health.nsw.gov.au | Children's Hospital at Westmead Clinical School, Faculty of Medicine and Health, University of Sydney | Sydney, Australia |
| Thomas | James | [james.thomas2@health.nsw.gov.au](mailto:james.thomas2@health.nsw.gov.au) | Department of Neurology, Liverpool Hospital | Sydney, Australia |
| Thomas | Terrence | terrence.thomas@singhealth.com.sg | Neurology Service Department of Paediatrics KK Women’s & Children’s Hospital Singapore | Singapore, Singapore |
| Thompson | Julia | juliathompsonneurology@gmail.com | Department of Neurology, Prince of Wales Hospital | Sydney, Australia |
| Trewin | Benjamin P | benjamin.trewin@sydney.edu.au | Translational Neuroimmunology Group, Kids Neuroscience Centre, Children's Hospital at Westmead, Sydney, New South Wales, Australia. | Sydney, Australia |
| Troedson | Christopher | christopher.troedson@health.nsw.gov.au | Children's Hospital at Westmead Clinical School, Faculty of Medicine and Health, University of Sydney | Sydney, Australia |
| Van der Walt | Anneke | anneke.vanderwalt@monash.edu | Multiple Sclerosis and Neuroimmunology Research Groups, Department of Neuroscience, Monash University | Melbourne, Australia |
| Vucic | Steve | steve.vucic@sydney.edu.au | Department of Neurology, Concord Repatriation General Hospital | Sydney, Australia |
| Wang | Justine | justine.wang@health.nsw.gov.au | Department of Neurology, St George Hospital | Sydney, Australia |
| Ware | Tyson | tyson.ware@ths.tas.gov.au | Department of Paediatrics, Royal Hobart Hospital | Hobart, Australia |
| Webster | Richard | richard.webster@health.nsw.gov.au | Department of Neurology, The Children's Hospital at Westmead | Sydney, Australia |
| Wei Lin | Ming | mingwei.lin@health.nsw.gov.au | Department of Clinical Immunology and Immunopathology, Institute of Clinical Pathology and Medical Research, Westmead Hospital, University of Sydney | Sydney, Australia |
| White | Owen | owen.white@monash.edu | Monash University | Melbourne, Australia |
| Wolfe | Nigel | Nigel.wolfe@health.nsw.gov.au | Department of Neurology, Blacktown Hospital | Sydney, Australia |
| Yeh | Wei | wei.yeh@monash.edu | Department of Neurology, Alfred Health | Melbourne, Australia |
| Yiannikas | Con | y.con@bigpond.com | Department of Neurology, Concord Repatriation General Hospital | Sydney, Australia |
| Yiu | Eppie M | eppie.yiu@rch.org.au | Department of Neurology, The Royal Children's Hospital Melbourne, Neurosciences Research, Murdoch Children’s Research Institute | Melbourne, Australia |
| Zhong | Michael | michael.zhong@monash.edu | Multiple Sclerosis and Neuroimmunology Research Groups, Department of Neuroscience, Monash University | Melbourne, Australia |
